# KNN-Based Machine Learning Classifier Used on Deep Learned Spatial Motion Features for Human Action Recognition

**DOI:** 10.3390/e25060844

**Published:** 2023-05-25

**Authors:** Kalaivani Paramasivam, Mohamed Mansoor Roomi Sindha, Sathya Bama Balakrishnan

**Affiliations:** 1 Department of Electronics and Communication Engineering, Government College of Engineering, Bodinayakanur 625582, Tamilnadu, India; 2 Department of Electronics and Communication Engineering, Thiagarajar College of Engineering, Madurai 625015, Tamilnadu, India; smmroomi@tce.edu (M.M.R.S.); sbece@tce.edu (S.B.B.)

**Keywords:** spatial motion cue, directed acyclic graph-based residual 2D CNN, deep learned feature, KNN classifier

## Abstract

Human action recognition is an essential process in surveillance video analysis, which is used to understand the behavior of people to ensure safety. Most of the existing methods for HAR use computationally heavy networks such as 3D CNN and two-stream networks. To alleviate the challenges in the implementation and training of 3D deep learning networks, which have more parameters, a customized lightweight directed acyclic graph-based residual 2D CNN with fewer parameters was designed from scratch and named HARNet. A novel pipeline for the construction of spatial motion data from raw video input is presented for the latent representation learning of human actions. The constructed input is fed to the network for simultaneous operation over spatial and motion information in a single stream, and the latent representation learned at the fully connected layer is extracted and fed to the conventional machine learning classifiers for action recognition. The proposed work was empirically verified, and the experimental results were compared with those for existing methods. The results show that the proposed method outperforms state-of-the-art (SOTA) methods with a percentage improvement of 2.75% on UCF101, 10.94% on HMDB51, and 0.18% on the KTH dataset.

## 1. Introduction

Recognizing human actions is a vital process in most computer vision applications, such as violence detection [1,2], surveillance video analysis [3], anomaly detection [4], video retrieval, video summarization [3], elder care monitoring, and emergency rescue operations [5]. Human action recognition is also applicable to robot-assisted surgical methods. A hand-gesture-based robot-assisted surgical method, with the support of augmented reality (AR), was proposed by Rong Wen [6]. In their approach, preoperative and intraoperative information was directly shown over a patient in a projector-based AR environment while a mobile surgical robot system executed predefined RF needle insertion plans. A Siamese-oriented region proposal network was presented for visual tracking application in [7]. An augmented surgical planning approach was proposed with model-section images of a real patient’s body and direct augmented interactivity. Through the projection, correction, and registration of surgical models, a projector–Kinect system was presented to create a surgical environment with spatial augmented reality directly on the patient’s body [8]. Joint similarity measures and an adjustable weight-based stereo-matching algorithm were proposed in [9]. These methods enhance the matching cost computation to better fit the color image of the heart’s soft tissue. The approach simultaneously enhances the adaptive weight by using the concept of graph cutting. In recent years, deep-learning-based human action recognition [10,11,12,13] has had increased attention in the field of computer vision due to its efficiency in understanding context based on an imitation of our visual cortex. There are 2DCNN-based methods that use a two-stream approach and LSTM networks, and there are 3D CNN-based methods for HAR. Although 3DCNN performed well on a temporal stream of video, it has its own limitations due to its intensive computational complexity. This prompted researchers to develop two-stream-based 2DCNN methods. Complexity still exists in the two-stream approach for traversing twice through the network. Therefore, a novel single stream, directed acyclic graph-based residual 2D CNN, also known as HARNet, has been proposed.

The proposed work is motivated by the information bottleneck theory [14], which argues that the objective of any supervised learning strategy is to extract and competently represent the significant information content in the input data that correspond to the output label. According to the interpretation of information theory of minimally sufficient statistics, input data need to be mapped in a maximally compressed format to the output label by preserving the information content as much as possible. The proposed work introduces a novel single-stream learning framework called HARLearning for the prediction of human action. The proposed method exploits the capability of CNN in learning a maximally compressed but informative representation by designing a customized, shallow, layered graph (residual CNN). The shallow network architecture was chosen to guarantee the optimal performance of the model, even if the training samples were of lesser quality, which will be helpful to recognize abnormal actions in surveillance videos, where the amount of abnormal action data is less comparable to that of normal actions. Alternative reasons are that shallow networks can be easily deployed on edge devices, as the structural simplicity and the number of convolution layers used in the shallow network are reduced, which leads to a reduced number of parameters, again diminishing computational complexity and training time. The term “layered graph-based CNN architecture” means having layers with multiple inputs or multiple outputs. This effectively means that information from one layer is directly landing on the next layer, with the exception of the regular flow. This is achieved by providing residual connections. The learned features have been used to train ML classifiers, such as support vector machine (SVM), decision tree (DT), linear discriminant analysis (LDA), naïve Bayes (NB), ensemble, and k-nearest neighbor (KNN), for recognizing human action. For human action recognition, temporal/motion information is important. Both spatial and temporal information are combined to construct a novel spatial motion cue. Spatial information is preserved by gray-level frames, which are combined with optical flow motion vectors in the band axis for including the temporal aspect.

**The major contributions are as follows:**

- The fusion of spatial and temporal cues, represented by intensity and optical flow vectors, respectively.
- The proposal of a single-stream shallow network—HARNet Architecture—for extracting deep learned action features.
- The proposal of a KNN-based machine learning framework for classifying up to 101 human actions.
- Experimentation and comparison with SOTA (state-of-the-art techniques) on a benchmark dataset.

The organization of the paper is as follows. Section 2 gives the literature related to the proposed work. Section 3 describes the proposed spatial motion feature learning framework. Section 4 depicts the experimental details of the network learning setup with results and discussion. Section 5 gives conclusions and future directions.

## 2. Related Work

Recently, deep learning networks have been widely used for analyzing events in videos. One such model was designed using ResNet50 to extract the important features of each frame of the input, followed by a recurrent neural network (ConvLSTM) for detecting any abnormal events [2]. A method using a combination of CNN and long short-term memory (LSTM) for classifying video in a smaller dataset was proposed in [15]. Three different variants of input were evaluated using Resnet-152 for encoding and decoding based on a three-layer LSTM. The first input was the RGB frames, the second was the optical flow, and the third was the combination of both RGB frames and optical flow. The hybrid deep learning network (HDLN) proposed in [16] has been used to extract the features from complex smartphone inertial data. Deep learning models can also be used to automatically recognize the activity of a single worker. A method integrating CNN with SVM and R-CNN was proposed in [17]. All of the extracted fine-grained action features were trained using an action-independent Gaussian mixture model (AIGMM). Spatio-temporal information was analyzed and the resemblances were preserved. The statistics of AIGMM, such as the mean, posteriors, and covariance were utilized to create kernels for finding the similarity [18].

Pedestrian attribute recognition (PAR) is very important in the computer vision techniques applicable for video surveillance systems. PAR-based methods have been implemented to provide a comparison between deep learning and conventional algorithms [19]. In order to recognize the group activity of individual interactions with relevant objects, a method based on skeletal information was proposed [20]. This method used the group interaction relational network (GIRN) to find the relationships among multiple modules and to find the interactions among them. To recognize human action from random views, a two-branch view action generation method built on auxiliary conditional GAN was proposed [21]. Using this approach, action samples were generated for the arbitrary view of human action and the view ranges of the action sequences were enlarged in the training set. A method based on the dual-camera framework was implemented to recognize and track non-driving activities (NDAs). This was achieved by mapping the driver’s gaze with a non-linear identification model using a deep learning algorithm [22]. Furthermore, skeleton-based human interaction recognition requires spatial, temporal, and interactive features. The dyadic relational graph convolutional network (DR-GCN) method was proposed for interaction recognition [23]. In recent works, transformers have been used for action recognition in videos. A comprehensive survey of approaches using vision transformers for action recognition is given in [24]. Action transformer (AcT), a straightforward, fully self-attentional architecture that outperforms more complex networks with a combination of convolutional, recurrent, and attentive layers, was presented in [25]. This approach took advantage of 2D posture representations during shorter temporal windows to reduce computing and energy demands.

## 3. Proposed Spatial Motion Feature Learning Framework

**Figure 1:**
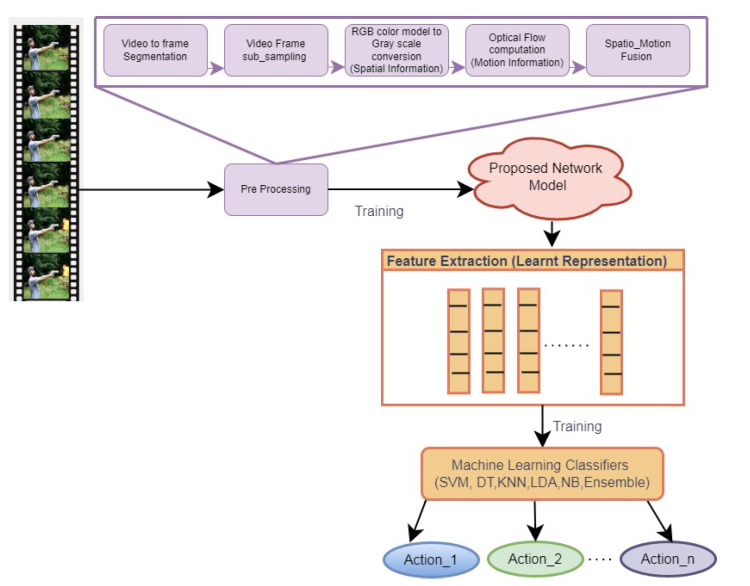
The proposed framework for learning deep spatial motion features.

The proposed spatial motion feature learning framework comprises three steps, namely preprocessing, the design of the neural network, and representation learning for HAR, as depicted in Figure 1. Preprocessing involves the fusion of spatial information with motion information. It is performed by concatenating the gray-scale form of video frames with motion vectors obtained from optical flow computation in the band axis.

### 3.1. Preprocessing

The input video data are segmented into frames, which are subsampled to reduce the redundancy. The motion vector is computed by finding the optical flow between the frames using the Horn and Schunk approach [26]. The intensity image is obtained from the RGB frame, which is concatenated with the horizontal and vertical vectors of optical flow. As the constructed input contains both spatial and motion information, it is named spatial motion fusion data.

### 3.2. Design of Proposed Network Model

**Figure 2:**
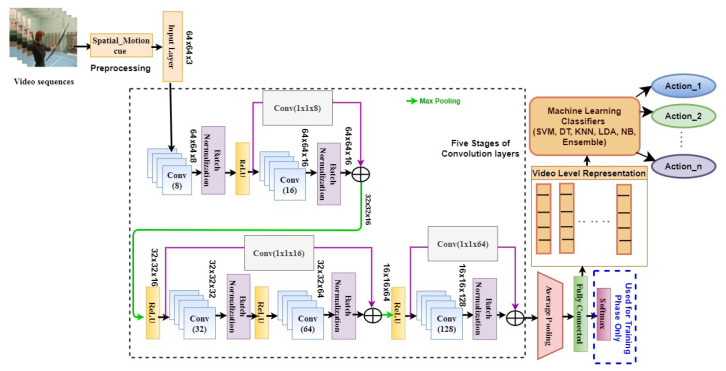
Architecture of the proposed HARNet.

The proposed network is constructed by stacking five stages of convolution layers, as shown in Figure 2. In the first stage, eight-channel convolution layers, each with kernel size of 3 and a single stride with the same padding, are used. Each convolution layer is followed by a batch normalization layer to provide normalization across mini-batches of data. The ReLU layer is used to retain the zeros and positive values of features alone.

The convolution layers in the following stages of the network have an increasing number of channels with a scaling factor of 2, but the size of the kernel is fixed at 3×3. Similarly, the stride and padding are the same as that of the first-stage convolution layer. The output feature maps of the ReLU layer of the first stage are added with the output feature maps of the batch normalization layer of the second stage via (1×1) convolution operation for skip connection. Then, the (2×2) max pooling layer is used to down-sample the size of the feature map to half of the value and to retain only the dominant features. Then, the ReLU layer is used in the second stage of the network. The output feature maps of this layer are added with the combined feature outputs of the third- and fourth-stage layers. The features of the second-stage ReLU are added with the fourth-stage batch normalization outputs. Then, the max pooling layer is used to downsize the dimensions of the features. Then, the ReLU layer is included, which is followed by the final fifth-stage convolution and batch normalization layers. The output feature maps of ReLU at the fourth stage are connected with output feature maps of batch normalization at the fifth stage. This is then followed by an average pooling layer and a fully connected layer. The fully connected layer receives an input with a size of 8×8×128 (8192), and the number of output nodes is chosen as the number of action classes used to train the network. The fully connected layer is used to extract high-level representations of data in a more condensed form. The final layer of the softmax layer is used only for learning the kernel weights of the proposed network during the training phase. Categorical cross entropy is used as the loss function while training the network model. After learning the HARNet kernel weights, the high-level features learned in the fully connected layer are extracted as representation. These features are used to train the k-nearest neighbor (k-NN) classifier for inferring the action characterized by the learned representation.

### 3.3. Information Bottleneck Principle

The information bottleneck principle is based on information theory, which is used to extract more significant contents contained by the random input variable X∈v, where *v* denotes preprocessed video data about the random output variable Y∈l, where *l* denotes the label of the output action category. Given their transition probabilities p(Y∣X), their joint probability distributions can be computed as

(1) p(X;Y)=p(Y∣X)p(X)

Significant average information is given by mutual information as

(2) $$I\left(X;Y\right)=\sum \sum \left[p\left(X;Y\right)\log_{2}\left(\frac{p(Y|X)}{p(Y)}\right)\right]$$

where statistical dependency between *X* and *Y* is assumed. As shown in Figure 2, each layer of the network operates on inputs obtained from the previous layer, which causes the neural network to form a Markov chain. Hence, data processing inequality (DPI) results from the fact that information about *Y* that is lost in one layer cannot be regained in the succeeding layers. According to the information theoretic learning principle for deep neural networks (DNNs), each layer in a DNN processes only inputs from previous layers. There is a loss of information in the consecutive layers (n≥m) as compared to the preceding layers, as depicted in the below equation. For any succeeding layer n≥m, it is given that

(3) $$I\left(Y;X\right)\geq I\left(Y;R_{m}\right)\geq I\left(Y;R_{n}\right)\geq I\left(Y;\hat{Y}\right)$$

where $R_{n}$ is the representation at a higher layer, $R_{m}$ is the representation at a lower layer, and $\hat{Y}$ is the predicted label for the true label *Y*. The equality in the above expression can be achieved if, and only if, each layer gives sufficient statistics for its input. Hence, it is necessary to obtain not only the most pertinent representation at each layer, but also the most compact representation of its input. Hence, a massive layered network may result in an information bottleneck. This limitation can be overcome by designing a shallow network. Each layer needs to try to enhance $I(Y;R_{n})$ while diminishing $I(R_{n-1};R_{n})$ as much as possible. This is achieved with the help of convolution neural network layers.

### 3.4. Classification

The features learned in a fully connected layer are extracted to train the machine learning classifiers. The efficacy of the proposed network in latent representation learning has been analyzed with various ML classifiers, such as support vector machine (SVM), decision tree (DT), k-nearest neighbor (KNN), linear discriminant analysis (LDA), naïve Bayes (NB), and ensemble.

- k-Nearest Neighbor classifier: k-NN is practically applicable for recognizing patterns of human actions, as it is non-parametric, which means that it does not assume the distribution of data. Hence, it works well in our proposed approach. The k-NN classifier stores all cases of training data and tries to classify test data based on similarity measures. Euclidean distance is considered as a similarity measure for finding the neighbors in our experiment. The number of neighbors included for classifying the test sample is one.
- Support Vector Machine: Complex data transformation is performed by SVM based on the chosen kernel function. With the help of those transformations, the separation boundary between data is maximized.
- Decision Tree: The decision tree is a systematic approach used for multi-class classification problems. A set of queries in relation to the features of a dataset is posed by DT. It is visualized using a binary tree. Data on the root node are again split into two different records that have different attributes. The leaves represent the classes of the dataset.
- Naïve Bayes: Bayes theorem is the basis of the naïve Bayes classification method. The naïve Bayes method is used because of the assumption that there is independence between every pair of features in the data.
- Linear Discriminant Analysis: The linear discriminant analysis (LDA) classification method is used in our experiments. It assumes that data of different classes are based on different Gaussian distributions. LDA uses the estimated probability that a piece of test data belongs to a particular class for classifying it. The class with the highest probability is predicted as the output class of the given sample.
- Ensemble: The adaptive boosting multi-class classification method is used as an ensemble aggregation approach for our experimentation. The number of learning cycles used in our experiments is 100, with the same learning rate for shrinkage.

In order to train the classifiers, the latent representations are retrieved from the training dataset. The classes of representations that were learned for the test dataset are then predicted using the trained models. The hyperparameters of the k-NN classifier are tuned while training machine learning classifier models with KTH, as shown in Table 1; the results of hyperparameter tuning experiments have also been listed.

## 4. Experimental Details on Network Learning Setup

The overall training regime of the HARNet is described in this section. Video sequences are preprocessed to obtain the fusion of spatial and motion information. The input to the network is downsized to 64×64×3. The convolution layer that comes after the input layer has weights that are initialized using the Glorot initializer, which helps to stabilize the training phase and shorten the training time. The kernel weights of size 3×3 are updated with a stochastic gradient descent. The first convolution layer has eight channels, and this number is doubled in the following convolution layers. Each kernel moves along its input with a single stride in all of the convolution layers of the network. The size of all max pooling layers in the model is 2×2 with a stride of 2. The hyperparameters of the proposed HARNet are tuned through experimentation by changing them in the linear range, as shown in Table 2. Following tuning process, the initial learning rate, the momentum, and the mini-batch size are set as 0.01, 0.5, and 32, respectively. The three-phase approach, comprising training, testing, and validation, is used to prevent the network from overfitting. The data split ratio used for implementing the three-phase approach was 70:15:15, and all experimentation was performed three-fold. The data split of 70:15:15 was carried out on a video-wise basis to provide a sufficient amount of video samples for training. The training set of video data was used to train the network model for learning kernel weights and validation data were used during training to improve the performance of the network. The testing set of video data was passed over the trained model to predict the action category of unseen data.

### 4.1. Datasets for HAR

Three standard HAR datasets considered for experimentally evaluating the performance of the proposed work are HMDB51, UCF101, and KTH. The various features of the datasets, such as the number of video clips, frame rate, number of action categories, challenges, and variations in data capture are listed in Table 3.

HMDB51 [27] is a large-scale human motion database containing 6849 videos of 51 action classes. Actions are categorized into five major groups, common body movement actions, common facial actions, body movement actions with an object, facial actions with an object, and human interactions with body movements. The UCF101 [28] dataset comprises 13320 videos under 101 action categories with enormous diversity in actions. There are large variations in the pose and appearance of objects in different scales, view points, and illumination conditions, potentially with camera motion or different cluttered backgrounds. The KTH dataset [29] contains 2391 video clips captured over a uniform background using a static camera at a frame rate of 25 frames per second. The dataset includes six actions, namely boxing, running, jogging, walking, hand clapping, and hand waving. Videos are captured in four different scenarios, indoors, outdoors, outdoors with different scales, and outdoors with different clothes.

### 4.2. Results and Discussion

To quantitatively assess the performance of the proposed network model, the standard evaluation metrics for assessing the accuracy of human action recognition are calculated for the overall model using Equation (4).

(4) $$Accuracy_{Model}=\frac{TP+TN}{\left(TP+TN+FP+FN\right)}$$

The other performance evaluation metrics of precision, recall, specificity, and F1-score are evaluated in order to compare the performance of different classification models, as well as to analyze the performance of the same model by varying different parameters [30]. The performance evaluation metrics of precision, recall, specificity, and F1-score have been defined for multi-class action recognition problems as an average of those metrics per class. The evaluation metrics per class are defined by assuming them to be a binary classification problem, such that the class under consideration is taken to be a positive case, and all other classes as negative cases, formulated using Equations (5)–(8).

(5) $$Precision_{PerClass}=\frac{TP}{\left(TP+FP\right)}$$

(6) $$Recall_{PerClass}=\frac{TP}{\left(TP+FN\right)}$$

(7) $$Specificity_{PerClass}=\frac{TN}{\left(TN+FP\right)}$$

(8) $$F1score_{PerClass}=\frac{\left(2*TP\right)}{\left[(2*TP)+FP+FN\right]}.$$

By taking the macro average, which is the arithmetic mean of the performance metric over each class, the evaluation metrics are computed for multiple classes.

#### 4.2.1. Evaluation on UCF101

The proposed network model was trained with preprocessed UCF101 video clips for categorizing 101 action classes. After learning the weights and latent representation of data, the features were extracted from the fully connected layer of the proposed network. The extracted features were used to train conventional ML classifiers for evaluating the efficacy of the network in learning latent representation. Human actions in the test data were predicted by the trained ML models with an average classification accuracy of 98.33% with LDA, 93.73% with NB, 93.04% with ensemble, 99.56% with DT, 99.98% with SVM, and 99.99% with KNN. A detailed classification report listing all of the standard multi-class performance metrics is given in Table 4. KNN yields the maximum values of 0.9999 for precision, 0.9990 for recall, unity for specificity, and 0.9999 for F1-score. From the values observed in Table 3, it is inferred that the proposed method works well on UCF101 data in classifying actions. Values for other metrics of precision, recall, specificity, and F1-score are also high irrespective of the ML classifier.

#### 4.2.2. Evaluation on HMDB51

Standard metrics such as accuracy, precision, recall, F1-score and specificity for the HMDB51 dataset are listed in Table 5. The proposed method recognizes human actions with an accuracy of 86.39% with LDA, 79.16% with NB, 77.97% with ensemble classifier, 88.01% with SVM classifier, 86.51% with DT, and 89.41% with KNN classifiers. From the values listed in Table 4, it reveals that the KNN classifier yields maximum values for various metrics, such as a precision of 0.8943, recall of 0.8933, specificity of 0.8999, and F1-score of 0.8938.

#### 4.2.3. Evaluation on KTH Dataset

The performance of the proposed approach had been experimentally evaluated on the untrimmed KTH dataset, which has six action classes. As this dataset contains frames with and without humans in the foreground, a preprocessing step of detecting people with the Gaussian mixture model was used. After detecting humans in the foreground, the frame was converted to a spatial motion cue, which was further processed by the network model for learning latent representation. The learned representation was used to train ML classifier models and then the test dataset was used to assess the prediction accuracy, precision, recall, specificity, and F1-score. Table 6 lists the values of the performance metrics computed on the KTH dataset. The proposed work yields an average classification accuracy of 95.93% with LDA, 95.76% with NB, 96.64% with ensemble, 96.50% with DT, 97.16% with SVM and the maximum performance with the KNN classifier of 97.49%. KNN results in a precision of 0.9667, recall value of 0.9623, specificity of 0.9951, and F1-core of 0.9644.

The results shown in Table 4, Table 5 and Table 6 indicate that the proposed method yields a high precision value irrespective of classifier. It implies that more positive predictions made by the proposed methodology are correct, which shows the efficiency of the proposed work in learning representations of human action. The high recall values reveal that most of the positive predictions made by the model are correct, out of all the actual positive samples. The high specificity values of all ML classifiers suggest that the trained models classify more negative samples in test data correctly. As the F1-score is the harmonic mean of precision and recall, a high F1-score demonstrates that the classifiers predict the test sample correctly by considering both false-positive and false-negative results. The high values of these metrics depict the strong robustness of the proposed representation learning for HAR.

#### 4.2.4. Results of the Ablation Study

**Figure 3:**
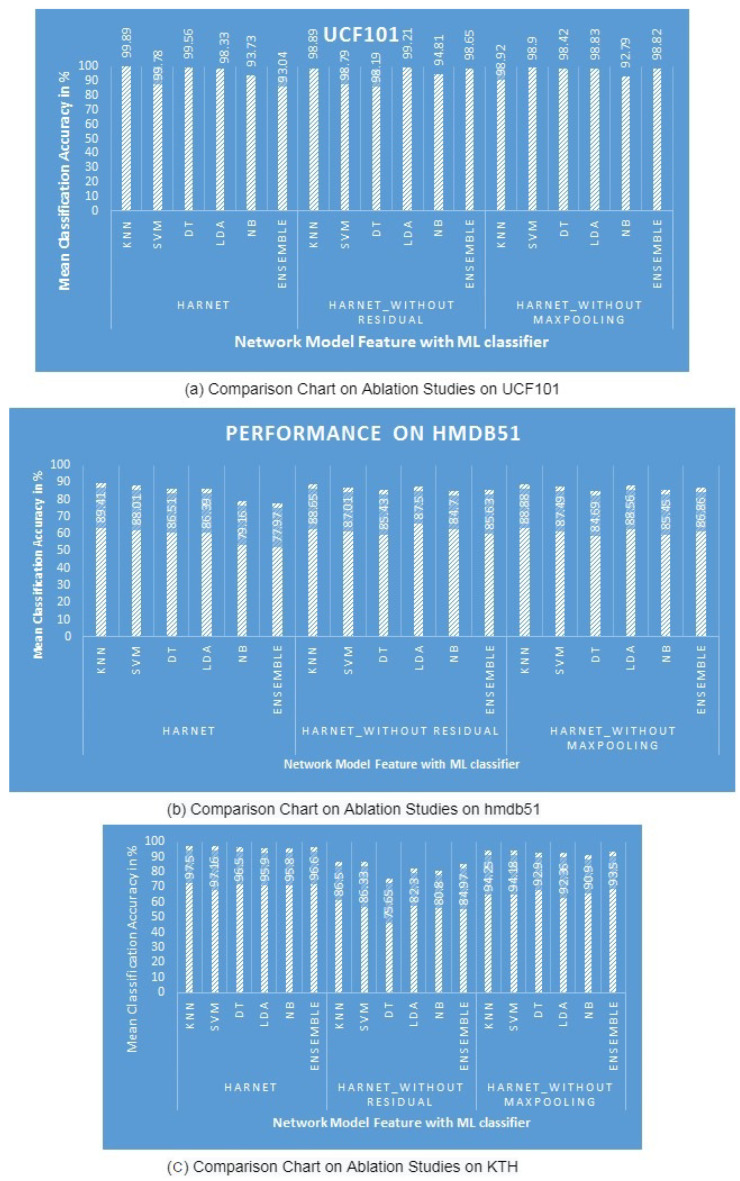
Comparison of the ablation study with six classifiers on three datasets.

The ablation study was conducted to determine the importance of each layer in influencing the performance of the proposed network model. It was implemented by removing certain layers from the proposed model; the experimental results are listed in Table 7. In the first study, to understand the importance of residual connections, the skip connections via 1×1 convolution and addition layers were removed, with the result being named HARNet_without Residual. The outcomes demonstrate that the removal of residual connections drops the maximum performance of the original model with the KNN classifier by 0.86% on HMDB51, 1% on UCF101, and 11.28% on KTH. In the second study, the max pooling layers alone were removed from the original, referred to as HARNet_without Maxpooling, by keeping the residual connections and addition operations to understand their contributions. It can be observed that the removal of max pooling layers reduces the performance of the proposed network model in learning representation by 0.59% on HMDB51, 0.97% on UCF101, and 3.33% on KTH, as shown in Figure 3. The results confirm that the residual connections and max pooling layers are significant in learning representation for human action recognition.

### 4.3. Comparison with State-of-the-Art Methods

The efficacy of the proposed network in representation learning has been analyzed and compared with other existing methods on the three benchmark datasets of UCF101, HMDB51, and KTH. Since state-of-the-art (SOTA) methods often employ a data split of 70:30, the proposed method also uses this split in order to compare with SOTA techniques. The results of action recognition experiments using the proposed network features classified by k-NN are compared with earlier studies, and are listed in Table 8, Table 9 and Table 10. It can be inferred from Table 8 that the proposed methodology increases performance on UCF101 over the existing approach [31] (Twostream) by 2.75%. Table 9 compares the performance of the proposed work with that of existing works on the HMDB51 dataset and shows that the mean prediction accuracy of the proposed method on HMMDB 51 is improved by 10.94% compared with the best-performing existing work [32]. The results on the KTH dataset are compared with those of existing works in Table 10. These results suggest that the proposed approach yields a 0.18% improvement in performance compared with the existing method [33]. The proposed HARNet features have also been trained with the two latest variants of k-NN, centroid displacement based on the k-Nearest Neighbor [34] and ensemble k-nearest neighbor based on centroid displacement (ECDNN) [35]. This experimentation shows that there is a minimal improvement in accuracy of 0.02% with CDNN and ECDD on UCF101, there is a considerable improvement in accuracy of 0.86% with CDNN and 1.36% with ECDNN on KTH, whereas a relatively lower accuracy (1.5% less with CDNN and 0.23% less with ECDNN) was found for HMDB51. The total number of learnable parameters in the proposed and existing deep neural networks are listed in Table 11. As the proposed network has only five 3×3 convolutional layers, three 1×1 convolution layers, and a fully connected layer with a number of action classes (N_c) times 8192 weights with N_c count of bias terms, the proposed HARNet model includes 525,779 kernel weight and bias terms. These weights are added along with 496 offset and scale parameters from the five stages of batch normalization layers, resulting in a total of 526,275 parameters. When compared with the best existing method [36], this is lower by 83.12%.

## 5. Conclusions and Future Directions

In this work, a representation learning model of HARLearning based on the information bottleneck principle is proposed. It was implemented by designing a novel directed acyclic graph-based residual CNN named HARNet. It was built by stacking convolution layers followed by a batch normalization layer and ReLU with max pooling layers after residual connection via (1×1) convolution. The network was trained for learning the latent representations of input and recognizing human actions. The fusion of spatial and motion information was performed by concatenating the form of the frame with optical flow vectors. Using the advantages of batch normalization and residual connections, the network was able to better understand the distinct features of human actions. The efficiency of the network in learning features to recognize actions with fewer parameters was achieved by the use of the max pooling layer and (1×1) convolution. The learned features were used to train ML classifiers. HARNet features with the KNN classifier yielded an improvement in performance in terms of accuracy by 2.75% on UCF101, 10.94% on HMDB51, and 0.18% on KTH compared with SOTA methods. The other metrics demonstrate the robustness of the proposed method on HAR with far fewer network parameters, which aids in implementing the proposed model on edge devices. The proposed work can be extended to unsupervised models for HAR to enable them to classify unseen data during surveillance. The model can be trained with a large new dataset, such as kinetics 700, in order to include more actions.

## Figures and Tables

**Table 1:** Tuning of hyperparameters of ML classifiers.

Model	Hyper Parameter Tuning				Accuracy (%) before Tuning	Accuracy (%) after Tuning
k-NN	Hyperparameter	K	Distance metric		95.58	97.49
	Tuning range	[1–100]	Euclidean, Cityblock, Minkowski, Chebychev, Hamming, Spearman, Cosine, Mahalanobis			
	Tuned value	1	Euclidean			
SVM	Hyperparameter	Box constraint	Coding	Kernel scale	95.93	97.16
	Tuning range	[0–1000]	1-vs-1, 1-vs-all	[0–1000]		
	Tuned value	1	1-vs-1	1		
DT	Hyperparameter	Minimum leaf size			94.26	96.50
	Tuning range	[1–300]				
	Tuned value	1				
LDA	Hyperparameter	Delta	Gamma		93.78	95.93
	Tuning range	[1 × 10^−6^, 1 × 10^−3^]	[0–1]			
	Tuned value	0	0.002			
NB	Hyperparameter	Distribution	Width		92.96	95.76
	Tuning range	Normal, kernel	[0–10]			
	Tuned value	Normal	-			
Ensemble	Hyperparameter	Number of learning cycles	Learning rate		94.05	96.64
	Tuning range	[1–400]	[0–1]			
	Tuned value	100	1			

**Table 2:** Tuning of hyperparameters of HARNet.

Model	Hyperparameter Tuning			
HARNet	Hyperparameter	Momentum	Initial learning rate	Mini-batch size
	Tuning range	[0–1]	[0.001–1]	[16–64]
	Tuned	0.5	0.01	32

**Table 3:** Various features of datasets.

Dataset	Features				
	Number of Video Clips	Frame Rate in Frames per Second	Number of Action Categories	Challenges	Variations in Data Capturing
HMBD51	6849	30	51	Camera movement	Camera view point, different video quality
UCF101	13320	25	101	Cluttered background, camera movement	Pose and appearance of object in varying scale, different illumination conditions and view points
KTH	2391	25	6	Presence of shadow, low-quality video	Scale variation, subjects with different clothes, indoors, outdoors

**Table 4:** Comparison of the influence of ML classifiers on UCF101.

ML Classifier	Accuracy	Precision	Recall	Specificity	F1-Score
LDA	98.33	0.9826	0.9834	0.9998	0.9826
NB	93.73	0.9392	0.9390	0.9994	0.9361
Ensemble	93.04	0.9114	0.8934	0.9993	0.8906
DT	99.56	0.9953	0.9951	1	0.9951
SVM	99.98	0.9998	0.9998	1	0.9998
KNN	99.99	0.9999	0.9990	1	0.9999

**Table 5:** Comparison of influence of ML classifiers on HMDB51.

ML Classifier	Accuracy in %	Precision	Recall	Specificity	F1-Score
LDA	86.39	0.8680	0.8573	0.8993	0.8620
NB	79.16	0.7911	0.7846	0.8978	0.7857
Ensemble	77.97	0.8260	0.7416	0.8975	0.7722
SVM	88.01	0.8819	0.8767	0.8996	0.8792
DT	86.51	0.8638	0.8596	0.8993	0.8615
KNN	89.41	0.8943	0.8933	0.8999	0.8938

**Table 6:** Comparison of influence of ML classifiers on KTH.

ML Classifier	Accuracy	Precision	Recall	Specificity	F1-Score
LDA	95.93	0.9561	0.9372	0.9918	0.9450
NB	95.76	0.9468	0.9382	0.9916	0.9420
Ensemble	96.64	0.9564	0.9524	0.9934	0.9543
DT	96.50	0.9546	0.9505	0.9931	0.9525
SVM	97.16	0.9628	0.9586	0.9944	0.9606
KNN	97.49	0.9667	0.9623	0.9951	0.9644

**Table 7:** Performance in the ablation study.

Model	ML Classifier	HMDB51	UCF101	KTH
HARNet	KNN	89.41	99.89	97.50
	SVM	88.01	99.78	97.16
	DT	86.51	99.56	96.50
	LDA	86.39	98.33	95.9
	NB	79.16	93.73	95.8
	Ensemble	77.97	93.04	96.6
HARNet_without Residual	KNN	88.65 (0.86% ↓)	98.89 (1% ↓)	86.50 (11.28% ↓)
	SVM	87.01	98.79	86.33
	DT	85.43	98.19	75.65
	LDA	87.50	99.21	82.3%
	NB	84.70	94.81	80.8%
	Ensemble	85.63	98.65	84.97%
HARNet_without Maxpooling	KNN	88.88 (0.59% ↓)	98.92 (0.97% ↓)	94.25 (3.33% ↓)
	SVM	87.49	98.90	94.18
	DT	84.69	98.42	92.90
	LDA	88.56	98.83	92.36
	NB	85.45	92.79	90.9
	Ensemble	86.86	98.82	93.5

**Table 8:** Comparison with existing works on UCF101.

Author	Method	Year	Accuracy (%)
Simonyan, K. and Zisserman, A. [37]	Two-stream (fusion by SVM)	2014	88.00
Du et al. [38]	C3D (Fine tuned from I380k)	2015	85.20
Wang, et al. [36]	TSN	2016	94.2
Qiu et al. [39]	Pseudo 3D	2017	93.70
Zhou et al. [40]	Mixed 3D/2D conv Tube (MiCT)	2018	88.90
Tran et al. [31]	R(2 + 1)D-RGB(Kinetics)	2018	96.80
Tran et al. [31]	R(2 + 1)D-TwoStream(Kinetics)	2018	97.30
Tu et al. [41]	ActionS-ST-VLAD	2019	95.60
Li et al. [42]	DANet	2020	86.70
Perrett et al. [43]	TRX	2021	96.10
Yongmei Zhang [5]	STFusionNet	2022	93.20
Chen [44]	2L-Attention-s3DResNet	2023	95.68
**Proposed**	**HARNet + KNN**	-	**99.98**

**Table 9:** Comparison with existing works on HMDB51.

Author	Method	Year	Accuracy (%)
Simonyan, K. and Zisserman, A. [37]	Two stream(fusion by SVM)	2014	59.40
Wang, et al. [36]	TSN	2016	68.50
Zhou et al. [40]	Mixed 3D/2D conv Tube (MiCT)	2018	63.80
Tran et al. [31]	R(2 + 1)D-RGB (Kinetics)	2018	74.50
Tran et al. [31]	R(2 + 1)D-TwoStream (Kinetics)	2018	78.70
Tu et al. [41]	ActionS-ST-VLAD	2019	71.40
Li et al. [42]	DANet-50	2020	54.30
Rehman, Inzamam [32]	3DCF+NFC	2021	82.55
Perrett et al. [43]	TRX	2021	75.60
Omi et al. [45]	Multi-Domain	2022	75.62
Chen [44]	2L-Attention-s3DResNet	2023	72.60
**Proposed**	**HARNet + KNN**	-	**91.58**

**Table 10:** Comparison with existing works on KTH.

Author	Method	Year	Accuracy (%)
Bregonzio et al. [46]	Appearance + distribution − MKL Fusion	2012	94.33
Shuiwang et al. [47]	3DCNN	2013	90.20
Cho [48]	Local motion + full motion	2014	89.70
Yao [49]	STB + Pool	2016	95.83
Zhang et al. [50]	SIFT + BoW + SVM	2018	94.69
Zhang et al. [33]	3D Deconvolution NN2	2020	97.40
Mishra [51]	FEA + RBF-SVM	2022	96.20
**Proposed**	**HARNet + KNN**	-	**97.58**

**Table 11:** Comparison with existing works in Parameters *.

Author	Method	Pretraining Dataset	Year	Parameters (M)
Simonyan, K. and Zisserman, A. [37]	Two stream	ImageNet	2014	25
Du et al. [38]	C3D	Kinetics400	2015	34.6
Wang, et al. [36]	TSN	ImageNet	2016	24.3
Qiu et al. [39]	Pseudo 3D	ImageNet /Kinetics400	2017	25.4
Zhou et al. [40]	Mixed 3D/2D conv Tube (MiCT)	Kinetics400	2018	50.2
Li et al. [42]	DANet	-	2020	36.26
Wang [52]	TDN	Kinetics400 + ImageNet	2021	52.3
Omi et al. [45]	Multi-Domain	Kinetics400	2022	32.02
Chen [44]	2L-Attention-s3DResNet	Kinetics400	2023	3.08
**Proposed**	**HARNET**	**HMDB51**	-	**0.52**

* As the datasets used in this experiment are different, this comparison is given for information only.

## Data Availability

The data are available in a publicly accessible repository. The data presented in this study are openly available in UCF101 at http://arxiv.org/abs/1212.0402, reference number [28], HMDB51 at https://doi.org/10.1007/978-3-642-33374-3, reference number [27], and KTH at https://www.csc.kth.se/cvap/actions/, reference number [29].
